# MEF-CAAN: Multi-Exposure Image Fusion Based on a Low-Resolution Context Aggregation Attention Network †

**DOI:** 10.3390/s25082500

**Published:** 2025-04-16

**Authors:** Wenxiang Zhang, Chunmeng Wang, Jun Zhu

**Affiliations:** School of Computer Engineering, Jinling Institute of Technology, Nanjing 211169, China; 2307050021@stu.jit.edu.cn (W.Z.); zhujun@jit.edu.cn (J.Z.)

**Keywords:** multi-exposure image fusion, multi-resolution, context aggregation attention network, guided filtering for upsampling

## Abstract

Recently, deep learning-based multi-exposure image fusion methods have been widely explored due to their high efficiency and adaptability. However, most existing multi-exposure image fusion methods have insufficient feature extraction ability for recovering information and details in extremely exposed areas. In order to solve this problem, we propose a multi-exposure image fusion method based on a low-resolution context aggregation attention network (MEF-CAAN). First, we feed the low-resolution version of the input images to CAAN to predict their low-resolution weight maps. Then, the high-resolution weight maps are generated by guided filtering for upsampling (GFU). Finally, the high-resolution fused image is generated by a weighted summation operation. Our proposed network is unsupervised and adaptively adjusts the weights of channels to achieve better feature extraction. Experimental results show that our method outperforms existing state-of-the-art methods by both quantitative and qualitative evaluation.

## 1. Introduction

With the development of digital image processing technology, multi-exposure image fusion technology has been widely used in many fields, such as photography, medical image processing, remote sensing monitoring, and so on. This technology aims to produce an image with rich detail and a wide dynamic range by fusing images with different exposures. Existing MEF methods can be divided into traditional methods and deep learning-based methods.

### 1.1. Traditional Methods

The traditional methods can be divided into spatial domain-based fusion methods, such as the block-based method [1] orthe pixel-based method [2], and transformation domain-based fusion methods, such as Laplacian pyramid-based methods [3,4]. Goshtasby [5] divided the image into d×d blocks and selected the blocks that maximized, then performed a simple weighted summation to calculate the final output image. However, blocks can overlap with different objects, causing ghosting. Ma et al. [1] further decomposed the image block into three components, signal strength, signal structure, and average strength, and processed them by patch strength and exposure, but this can introduce halo artifacts around the boundary. The multi-scale decomposition based methods have been widely used in the field of image fusion. For example, Ben et al. [6] proposed a multi-scale framework fusion method based on biorthogonal wavelet transform and constructed composite wavelet coefficients based on information theory, but the method is not designed specifically for multi-exposure image fusion tasks. Burt et al. [7] proposed an efficient multi-scale image decomposition method based on the Laplacian pyramid, which greatly influenced the development of multi-exposure image fusion. Mertens et al. [4] proposed to construct weight maps by contrast, saturation, and exposure. Then the Laplacian pyramid of each input image is weighted with the Gaussian pyramid of its corresponding weight map to obtain the final fusion image. Li et al. [3] proposed a fast multi-exposure image fusion method based on structural patch decomposition, which effectively reduced the halo artifacts of edge attachments through multi-scale decomposition. Li et al. [8] used a method based on quadratic optimization to obtain fine details but ignored the natural visual quality. Liu et al. [9] used the method based on dense scale-invariant feature transform for feature extraction and a weight term scheme for exposure fusion. Although it can deal with the image ghost, the complexity is high. Wang et al. [10] proposed a multi-scale exposure fusion method in YUV space with a simple detail enhancement component, which is difficult to recover enough details when dealing with complex HDR scenes.

In summary, traditional exposure fusion methods based on spatial domain or transform domain rely on certain feature fusion rulers, which cannot fully capture the over-exposed or under-exposed information of complex scenes, resulting in unsatisfactory visual quality.

### 1.2. Deep Learning Based Methods

In recent years, the rise of deep learning technology has brought new solutions in the field of multi-exposure image fusion [11,12,13]. In particular, convolutional neural networks (CNNs) are widely used for fusion due to their respectable performance in feature extraction and image representation. DeepFuse [14] used a CNN to fuse Y channels of input images and MEF-SSIM [15] as a reference metric, but it cannot extract the features well because of the simple model. Qi et al. [16] proposed an unsupervised multi-exposure fusion network based on CNN, which uses the structural similarity index and the no-reference gradient fidelity term to construct the loss function. Compared with DeepFuse, this method can fuse three or more source image sequences and achieves better results in terms of structure, color, and texture. However, more parameters are introduced, and the efficiency of the algorithm is low. U2Fusion [17] used the gradient of depth features to ensure the similarity between the fused image and the input images but ignored the specific consideration of different fusion tasks. MEF-Net [18] generated the weight maps by receiving the low-resolution version of the input images, which reduced the computational cost. However, the global structure information is not considered. IFCNN [19] extracted the feature information of the image through the convolution layer and then constructed the corresponding fusion rules according to the type of the source image to fuse the feature information. Finally, the fused features were reconstructed to obtain the final fusion image. Cai [20] used 13 multi-exposure fusion methods to generate fused images and selected the best-quality image as the reference image. Then, CNN was used to train the constructed dataset so as to enhance the contrast of the single-exposure image. This method effectively solves the problem of artifacts, but it cannot reconstruct the saturated region. TransMEF [11] designed a self-supervised, multi-task learning network model based on Transformer architecture to capture long-distance dependence information in images and combined with CNN to extract source image features. However, the public dataset MS-COCO [21] used in TransMEF is mainly suitable for object detection and segmentation tasks, but the dynamic range and texture information of the images in this dataset are not suitable for the MEF task. Moreover, the algorithm cannot recover the color information of the image accurately. DPE-MEF [12] used a network including one feature extraction module and one color enhancement module, but it ignored the importance of different channels so it could not extract enough feature information. MEF-LUT [22] encoded the weights of exposure images into a one-dimensional lookup table for MEF tasks, but the fusion quality is not satisfactory. Xu et al. [13] introduced GAN (Generative Adversarial Network) into the multi-exposure image fusion network for the first time. The generator fused the input multi-exposure images to generate the fused image, and the discriminator distinguished the fused image to the real image. However, most real images used in this method are generated from other fusion methods, so the performance of the GAN-based method is highly dependent on the quality of the generated real images.

Although the existing multi-exposure image fusion methods based on CNN have made certain progress, they still face problems such as the loss of details in extremely exposed regions and the lack of rich global image information.

In order to solve the above problem, researchers explored multi-exposure image fusion methods based on attention mechanisms [23]. Hu et al. [24] proposed the channel attention mechanism. By calculating the importance score of each channel, channel attention helps the network focus on the most important features, thereby improving the performance of the model. There are some other attention mechanisms applied in the field of image fusion. For example, STFNet [25] designed a transformer-based feature fusion network with the self-attention module and salient cross-attention module for the infrared and visible image fusion task, which obtains the pixel-level global dependence and improves the feature representation ability of the network. Xie et al. [26] proposed an attention mechanism similar to soft attention maps when dealing with the task of overexposed infrared and visible image fusion. However, the above attention mechanism based methods [25,26] for the infrared and visible images are not completely applicable to our multi-exposure fusion task because the multi-exposure images contain both extremely overexposed and underexposed areas.

**Figure 1:**
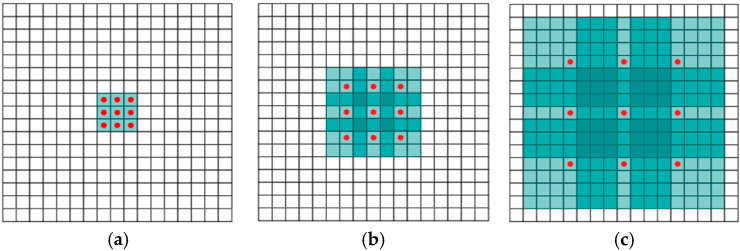
The receptive field at different dilation rates. Take a 3×3 convolution as an example. The colored region in (**a**) is the receptive field size with the dilation factor set to 1, and its size is 3×3. The colored region in (**b**) is the receptive field size with the dilation factor set to 2, and its size is 7×7. The colored region in (**c**) is the receptive field size with the dilation factor set to 4, and its size is 15×15.

Inspired by Ma [18], Hu [24], and Chen [27], we introduce the channel attention mechanism into the context aggregation network (CAN) [28] on the basis of the method [29] to improve the feature extraction ability of the model. CAN [28] mainly expands the receptive field by dilated convolution and aggregates multi-scale context information without changing the image resolution so as to enhance the feature extraction ability of the network for the source image. Figure 1 shows the receptive field at different dilation rates.

In this paper, we propose a multi-exposure image fusion method based on a context aggregation attention network called MEF-CAAN to predict the weight maps. The network can adaptively adjust the weights of different feature channels so as to extract better features for fusion. In addition, we adopt a low-resolution network to reduce computational complexity and increase processing speed. The main contributions of this paper can be summarized as follows:

1. We design an attention mechanism based on MEF-CAAN to effectively enhance the feature extraction ability of the network by focusing on the more important features and information of the source images, which achieves informative and visually natural fusion results.

2. Our CAAN and GFU modules expand the receptive field by dilated convolution, aggregate multi-scale context information, and generate the high-resolution weight maps by upsampling with input images guided, which jointly extract global features and preserve high-frequency information well.

## 2. Methodology

**Figure 2:**
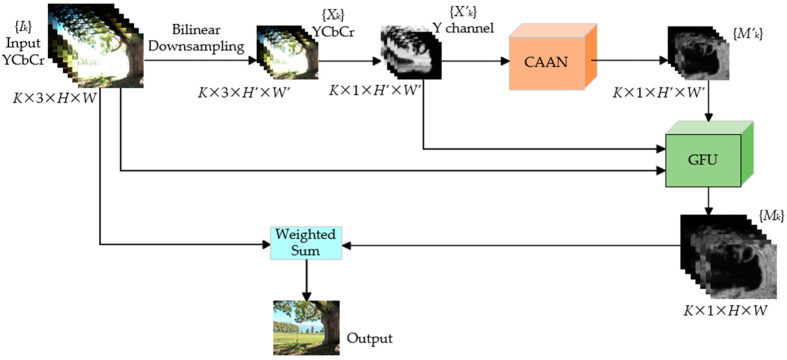
Our network structure diagram.

As shown in Figure 2, given the well-aligned input multi-exposure images $\left\{I_{k}\right\},k=1,\ldots ,K,I_{k}\in R^{3\times H\times W}$, we firstly downsample them to the low-resolution version $\left\{X_{k}\right\},X_{k}\in R^{3\times H^{\prime }\times W^{\prime }}$, then feed only Y channel to CAAN to get the low-resolution weight map $\left\{M_{k}^{\prime }\right\}$ because the Y channel has the most impact on the visual effect, which can also reduce the complexity of the model. We take $I_{k}$, $X_{k}^{\prime }$ and $M_{k}^{\prime }$ as inputs to GFU, to generate a high-resolution weight map $\left\{M_{k}\right\}$. Finally, the output image is obtained by weighted summation of $I_{k}$ and $M_{k}$.

### 2.1. Context Aggregation Attention Network (CAAN)

**Figure 3:**
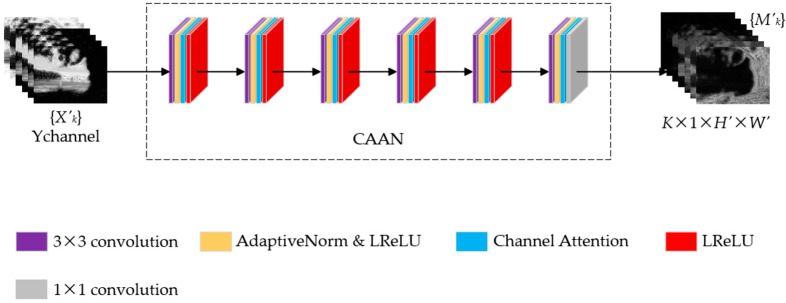
Network structure of CAAN.

Our approach takes CAN as the initial structure and introduces a channel attention mechanism. The network adopts dilated convolution to increase the receptive field without changing the spatial resolution. It aggregates context information at multi-scales by stacking convolutional layers with different dilation rates for better feature extraction. By learning the weights between different channels, the channel attention mechanism enhances the attention to more important channel features and suppresses less important channel features. In CAAN, multi-scale features are obtained and weighted at the channel level by the channel attention module. This enables the network to simultaneously capture multi-scale and channel-level features. The detailed structure of the CAAN is shown in Figure 3. The input to the network is a low-resolution version of the input sequence $\left\{X_{k}^{\prime }\right\}$ of any number of images, and the output is a low-resolution version of the weight map $\left\{M_{k}^{\prime }\right\}$.

The CAAN network has six layers, including five convolutional layers and one output layer. The feature maps of all the layers have the same resolution as the input image. The 3×3 size convolution kernels are used for all five convolution layers, and the dilation rate of each layer is set as 1, 2, 4, 8, and 1, respectively. The receptive field corresponding to each dilation rate is 3×3, 7×7, 15×15, 31×31, and 33×33, respectively. The 1×1 size convolution kernel is used for the output layer. The dilation rate is set to 1. We set the width of the output channels of each convolutional layer to 24, aiming to balance the feature extraction ability and computational complexity. Features are extracted with a convolution layer, and then adaptive normalization is used after the convolution layer. We use adaptive normalization, as in (1), to adjust parameters according to different input data, which has better flexibility. Besides, it can reduce the gradient explosion problem so that the model training can converge faster.

(1) $$F_{an}\left(T\right)=\alpha_{n}T+\beta_{n}IN\left(T\right),$$

where $\alpha_{n},\;\beta_{n}\in R$ are learnable weight parameters, T is the intermediate representation, and $IN\left(\cdot \right)$ represents the instance normalization operation.

**Figure 4:**
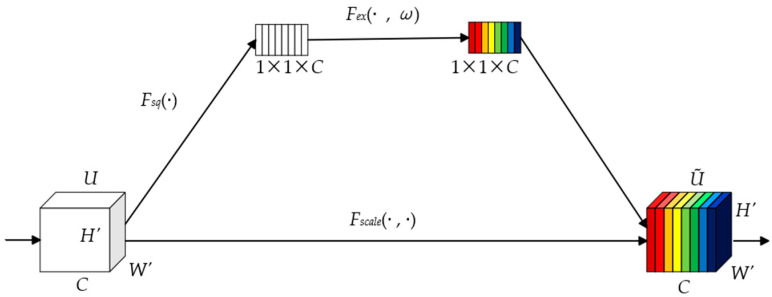
Channel attention network structure in CAAN.

In each convolutional layer, we introduce the channel attention mechanism to enhance the feature extraction ability of the network. As shown in Figure 4, the channel attention mechanism module first performs a squeeze operation on the input feature and uses the global average pooling method to encode the channel spatial features into global features. The calculation formula is as follows:

(2) $$z_{c}=F_{sq}(U)=\frac{1}{H^{\prime }\times W^{\prime }}\sum_{i=1}^{H^{\prime }}\sum_{j=1}^{W^{\prime }}U(i,\;\;j),$$

where i and j represent the pixel coordinates, $F_{sq}$ is the squeeze operation, and U is the feature input with the dimension number C.

**Figure 5:**
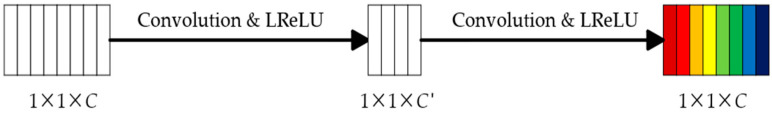
The detailed structure of $F_{ex}(\cdot ,\omega )$ operation.

The excitation operation is then applied to the global feature $z_{c}$, as shown in Figure 5, which consists of two convolutional layers and two activation functions. The excitation operation enables the network to learn the relationship between channels and also obtains the weights of different channels. The excitation operation is calculated as follows:

(3) $$s_{c}=F_{ex}\left(z_{c},\;\;\omega \right)=\sigma \left(g\left(z_{c},\;\;\omega \right)\right)=\sigma \left(\omega_{2}\sigma \left(\omega_{1}z_{c}\right)\right),$$

where $\omega_{1},\;\omega_{2}\in R^{\frac{C}{r}\times C}$ denotes that the dimension is $\frac{C}{r}\times C$, r is the scaling factor, and σ denotes the LReLU activation function. Finally, the operation $F_{scale}(\cdot ,\cdot )$ is performed, and each feature map is multiplied by its assigned weight to obtain the feature map $\tilde{U}$ processed by the channel attention mechanism. The formulation is as follows:

(4) $$\tilde{U}=F_{scale}(U,\;\;s_{c})=U\cdot s_{c},$$

We use LReLU as the activation function. The output layer generates the weight map $\left\{M_{k}^{\prime }\right\}$ using 1×1 convolution.

(5) $$F_{LReLU}(T)=max(\psi T,T),$$

where ψ is a fixed parameter during training.

### 2.2. Guided Filtering for Upsampling (GFU)

The output of CAAN is a low-resolution version of the weight map $\left\{M_{k}^{\prime }\right\}$. We apply guided filtering for upsampling to adjust $\left\{M_{k}^{\prime }\right\}$ back to $\left\{M_{k}\right\}$. GFU takes the source image sequence $\left\{I_{k}\right\}$, low-resolution version $\left\{X_{k}^{\prime }\right\}$, and low-resolution weight map $\left\{M_{k}^{\prime }\right\}$ as input, and the output is the high-resolution weight map $\left\{M_{k}\right\}$. Formalize as:

(6) $$W_{k}=GFU(I_{k},{X^{\prime }}_{k},{M^{\prime }}_{k}),$$

An important assumption of the guided filter is that there is a linear relationship between the input guided map $I_{i}$ and the output map $q_{i}$, which is expressed as follows:

(7) $$q_{i}=a_{k}I_{i}+b_{k},\;\forall i\in \omega_{k},$$

where $a_{k}$ and $b_{k}$ denote constant parameters and $b_{k}$ denotes the window centered at k. Guided filtering introduces the concept of noise; that is, the output image $q_{i}$ is the denoised image of the input image $p_{i}$.

(8) $$q_{i}=p_{i}-n_{i},$$

where n denotes the noise and i denotes the pixel index. In order to minimize the gap between the output of the fitted function $q_{i}$ and the true value $p_{i}$, the following equation is minimized:

(9) $$E(a_{k},b_{k})=\sum_{i\in \omega_{k}}\left({\left(a_{k}I_{i}+b_{k}-p_{i}\right)}^{2}+\epsilon a_{k}^{2}\right),$$

where ϵ is the regularization parameter that prevents a from being too large. We use $M_{k}^{\prime }$ as the input image for the guided filter and $X_{k}^{\prime }$ as the guided image.

### 2.3. Loss Function

We adopt the structural similarity index based MEF metric MEF-SSIM [15] as the loss function, which evaluates the quality of the fused image by considering intensity, contrast and structure information. Each image patch $x_{k}$ is determined by the following equation:

(10) $$x_{k}=\left\|x_{k}-\mu_{x_{k}}\right\|\cdot \frac{x_{k}-\mu_{x_{k}}}{\left\|x_{k}-\mu_{x_{k}}\right\|}+\mu_{x_{k}}\;=\left\|{\tilde{x}}_{k}\right\|\cdot \frac{{\tilde{x}}_{k}}{\left\|{\tilde{x}}_{k}\right\|}+\mu_{x_{k}}\;=c_{k}\cdot s_{k}+l_{k},$$

where $\left\|\cdot \right\|$ denotes the $l_{2}$-norm. $c_{k}=\left\|{\tilde{x}}_{k}\right\|$, $s_{k}=\frac{{\tilde{x}}_{k}}{\left\|{\tilde{x}}_{k}\right\|}$, and $l_{k}=\mu_{x_{k}}$ represent the contrast, structure, and intensity of $x_{k}$, respectively.

The desired contrast of the fused image patch is the maximum contrast in $\left\{x_{k}\right\}$.

(11) $$\hat{c}=\underset{1\leq k\leq K}{\max}c_{k},$$

The desired structure of the fused image patch is computed by weighted summation as follows:

(12) $$\hat{s}=\frac{\bar{s}}{\left\|\bar{s}\right\|},\;where\;\bar{s}=\frac{\sum_{k=1}^{K}\omega_{s}({\tilde{x}}_{k})s_{k}}{\sum_{k=1}^{K}\omega_{s}({\tilde{x}}_{k})},$$

where $\omega_{s}(\cdot )={\left\|\cdot \right\|}_{\infty }$ is an $l_{\infty }$-norm weight function.

The desired intensity of the fused image patch is defined by the following equation:

(13) $$\hat{l}=\frac{\sum_{k=1}^{K}\omega_{l}(\mu_{k},\;l_{k})l_{k}}{\sum_{k=1}^{K}\omega_{l}(\mu_{k},l_{k})},$$

where $\omega_{l}(\cdot )$ is a weight function of the global mean intensity $\mu_{k}$ of $X_{k}$ and the local mean intensity $l_{k}$ of $x_{k}$. $\mu_{l}(\cdot )$ is specified by a two dimensional Gaussian profile

(14) $$\omega_{l}(\mu_{k},l_{k})=exp(-\frac{{\left(\mu_{k}-\tau \right)}^{2}}{2\sigma_{g}^{2}}-\frac{{\left(l_{k}-\tau \right)}^{2}}{2\sigma_{l}^{2}}),$$

where $\sigma_{g}$ and $\sigma_{l}$ are two photometric spreads, set to 0.2 and 0.5, respectively. τ=128 represents the mid-intensity value for an 8-bit sequence.

The quality measure of each local image patch is determined by the following equation:

(15) $$S(\{x_{k}\},y)=\frac{\left(2\mu_{\hat{x}}\mu_{y}+C_{1})(2\sigma_{\hat{x}y}+C_{2}\right)}{\left(\mu_{\hat{x}}^{2}+\mu_{y}^{2}+C_{1})(\sigma_{\hat{x}}^{2}+\sigma_{y}^{2}+C_{2}\right)},$$

where $\mu_{\hat{x}}$ and $\mu_{y}$ denote the mean intensities of the desired patch and the fused patch, respectively. $C_{1}$ and $C_{2}$ are two constants to avoid zero denominators. $\sigma_{\hat{x}}$ and $\sigma_{y}$ denote the variances of the local patches $\hat{x}$ and y, respectively. $\sigma_{\hat{x}y}$ denotes the covariance of the local patches $\hat{x}$ and y. The overall quality measure of the fused image is obtained by averaging all image patches by:

(16) $$MEF\text{-}SSIM(\{X_{k}\},Y)=\frac{1}{M}\sum_{i=1}^{M}S(\{R_{i}X_{k}\},R_{i}Y),$$

where $R_{i}$ is the i-th patch of the image. The value of MEF-SSIM ranges from 0 to 1, with higher values indicating higher quality of fusion. Note that we only focus on the Y component optimized by the training, and the weighted summation method is used for Cb and Cr channels. Finally, YCbCr is converted to RGB as the output image.

### 2.4. Training

We selected 690 static sequences from Prabhakar [14], Cai [20], Endo [30], Fairchild [31], and Zeng [32] as our dataset. These sequences include kinds of scenes, both indoor and outdoor. We selected 600 sequences for training and the remaining 90 sequences for testing. We trained after resizing them to 512 × 512. Adam was used as the optimizer for training, and the learning rate was set to 10^−4^. The training epoch was set to 100. The parameter r of LReLU was set to 0.2. The radius and regularization parameters of the guided filter were set to 1 and 10^−4^, respectively.

## 3. Experiments

**Figure 6:**
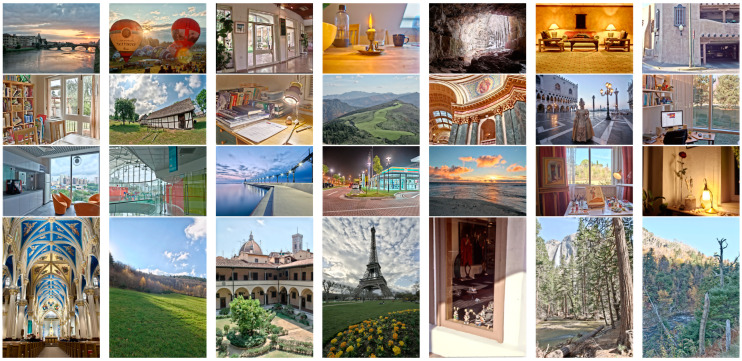
Some fused images by our method.

In this section, we compare our method with seven state-of-the-art methods by both subjective observation and objective quality measurement, including four traditional methods MEF09 [4], DSIFT [9], DEM [10], and FMMEF [3], and three deep learning based methods MEF-Net [18], DPE-MEF [12], and MEF-LUT [22]. In addition, we also compare the running time with the three deep learning-based methods. Some fused images by our method are shown in Figure 6.

### 3.1. Subjective Observation Comparison

**Figure 7:**
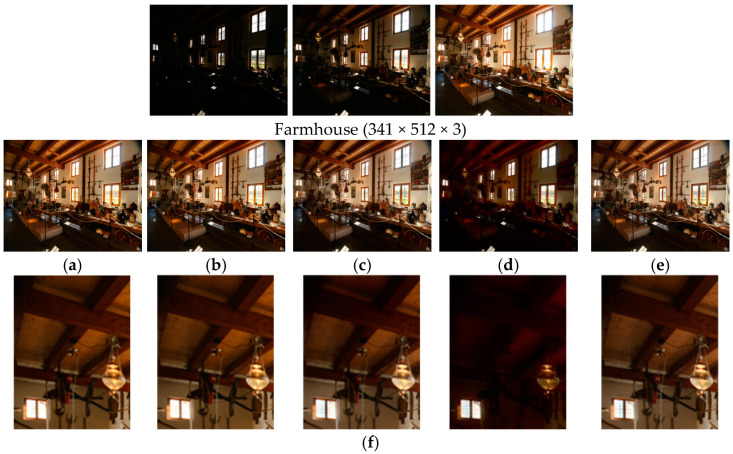
Comparison with other exposure fusion methods on the set ‘Farmhouse’. (**a**) MEF09 [4], (**b**) DEM [10], (**c**) MEF-Net [18], (**d**) MEF-LUT [22], (**e**) MEF-CAAN, (**f**) from left to right are partially enlarged images of a, b, c, d, and e, respectively.

Figure 7 shows the comparison results on the set ‘Farmhouse’. There are underexposed areas indoors and overexposed areas outside the window in this scene. It is very challenging to recover details in these areas because of their extreme exposure conditions. Among the five compared algorithms, MEF-LUT [22] obtained the worst fusion result with detail loss, significant artifacts, and color bias. MEF09 [4] and DEM [10] both lose overexposed detail information outside the windows, and the lines on the windows can hardly be seen. MEF-Net [18] has a certain color bias and insufficient brightness on the underexposed house ceiling. Our method achieves the best fusion result in terms of both detail recovery and overexposure suppression in the extremely exposed areas.

**Figure 8:**
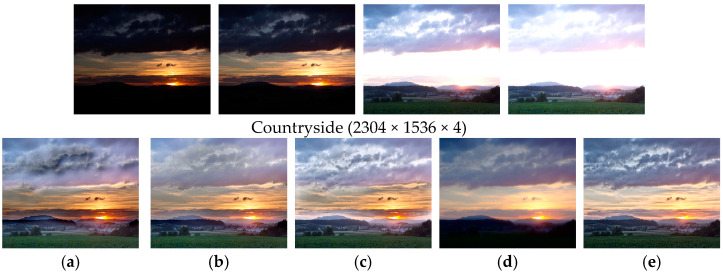
Comparison with other exposure fusion methods on the set ‘Countryside’. (**a**) MEF09 [4], (**b**) DEM [10], (**c**) MEF-Net [18], (**d**) MEF-LUT [22], (**e**) MEF-CAAN.

Figure 8 shows the comparison results on the set ‘Countryside’. There are clouds in the sky with rich texture information, overexposed sunlight, and underexposed grassland and mountains in this scene. MEF09 [4] suffers from significant halo artifacts and color bias in the sky. DEM [10] has low contrast on the boundary of the white clouds and blue sky, and the detailed information is lost. MEF-Net [18] also has obvious halos in the overexposed and underexposed transition areas, and the overall color recovery of the scene is not natural. MEF-LUT [22] lacks detailed information in both underexposed and overexposed areas. In this very challenging scene, our method recovers overexposed and underexposed details and natural colors of the scene.

**Figure 9:**
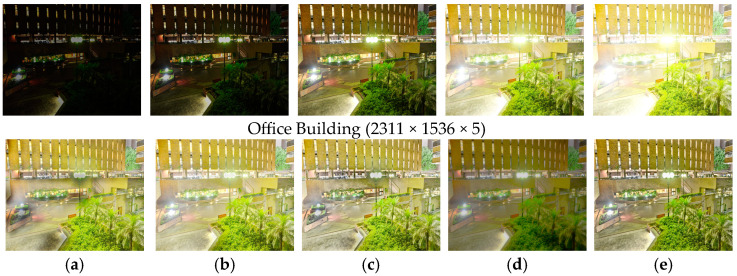
Comparison with other exposure fusion methods on the set ‘Office Building’. (**a**) MEF09 [4] (**b**) DEM [10] (**c**) MEF-Net [18] (**d**) MEF-LUT [22] (**e**) MEF-CAAN.

Figure 9 shows the comparison results on the set ‘Office Building’. There are extremely exposed areas, such as streetlights, street ground, and building walls, in this night scene. MEF09 [4] has obvious halo artifacts around the streetlights and on the ground, and the tree color is over-saturated. DEM [10] loses significant details around the streetlights and building walls. MEF-Net [18] also produces color bias in the overexposed and underexposed transition regions, such as around streetlights and ground. MEF-LUT [22] loses detailed information seriously. Our method recovers rich details and natural color in these areas, which is highly consistent with visual perception for night scenes.

**Figure 10:**
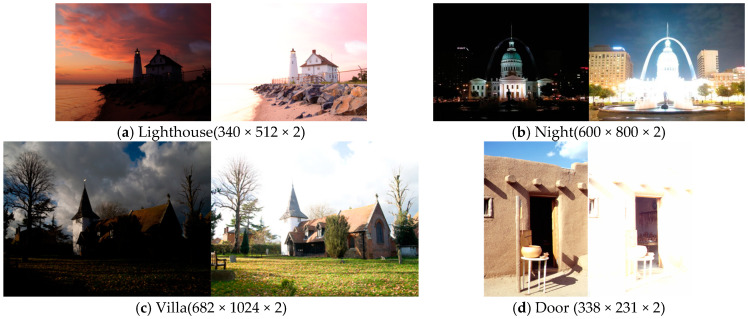
Representative testing sets with height, width, and the number of images.

The input of our method can be any resolution and any number of multi-exposure images, but in some existing multi-exposure image fusion algorithms, such as DSIFT [9] and FMMEF [3], the number of input images can only be two. Therefore, for a fair comparison, we also compare the fusion results of some testing sets with two input images, as shown in Figure 10.

**Figure 11:**
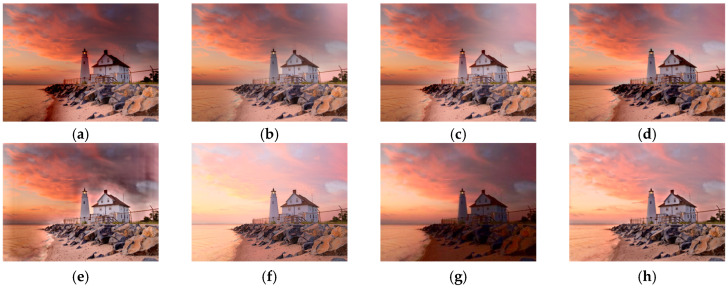
Comparison with other exposure fusion methods on the set ‘Lighthouse’. (**a**) MEF09 [4] (**b**) DSIFT [9] (**c**) DEM [10] (**d**) FMMEF [3], (**e**) MEF-Net [18] (**f**) DPE-MEF [12] (**g**) MEF-LUT [22] (**h**) MEF-CAAN.

Figure 11 shows the comparison results on the set ‘Lighthouse’ with a lighthouse at the seaside and the sunset in the background. MEF09 [4] has a slightly dark color in the sky and fewer details on the seaside stones and the roof. The colors and transitions of DSIFT [9], DEM [10], and FMMEF [3] for the light and shade are slightly not natural enough. At the same time, FMMEF [3] loses details on the stones. MEF-Net [18] has obvious halo artifacts around the house and in the sky, which affects the visual quality. Although DPE-MEF [12] has a certain color enhancement, the details of the clouds and water surface are seriously lost. MEF-LUT [22] also loses details and colors seriously.

**Figure 12:**
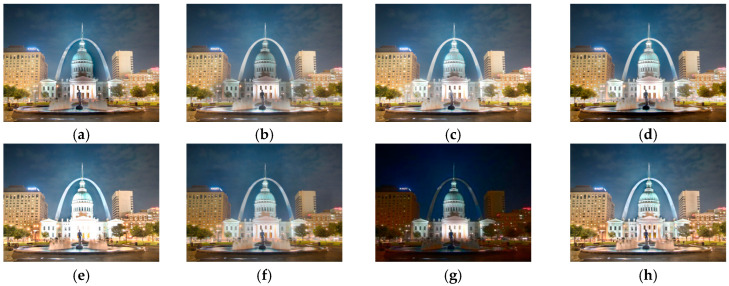
Comparison with other exposure fusion methods on the set ‘Night’. (**a**) MEF09 [4] (**b**) DSIFT [9] (**c**) DEM [10] (**d**) FMMEF [3], (**e**) MEF-Net [18] (**f**) DPE-MEF [12] (**g**) MEF-LUT [22] (**h**) MEF-CAAN.

Figure 12 shows the comparison results on the set ‘Night’. There are obvious halo artifacts around the buildings of MEF09 [4], DSIFT [9], DEM [10], and FMMEF [3], and at the same time, FMMEF [3] is dark on the ground, resulting in loss of details. MEF-Net [18] loses details in the overexposed building area. The image fused by DPE-MEF [12] is blurred, and the details are not rich enough. The image fused by MEF-LUT [22] is very dark and loses lots of details in the sky and ground.

**Figure 13:**
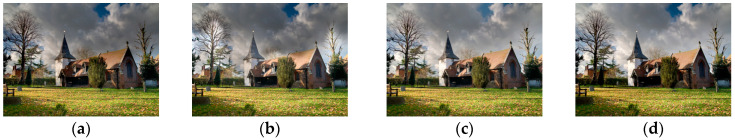

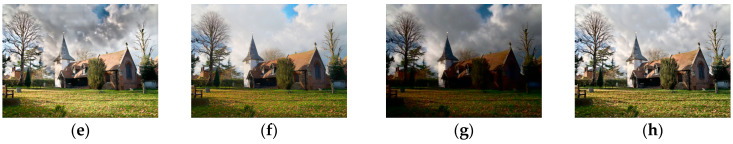
Comparison with other exposure fusion methods on the set ‘Villa’. (**a**) MEF09 [4] (**b**) DSIFT [9] (**c**) DEM [10] (**d**) FMMEF [3], (**e**) MEF-Net [18] (**f**) DPE-MEF [12] (**g**) MEF-LUT [22] (**h**) MEF-CAAN.

Figure 13 shows the comparison results on the set ‘Villa’. The fusion images of MEF09 [4], DSIFT [9], DEM [10], MEF-Net [18], and FMMEF [3] all have significant dark shadows in the clouds. DPE-MEF [12] loses detailed information inside the clouds, and MEF-LUT [22] also seriously loses details on the houses and trees.

**Figure 14:**
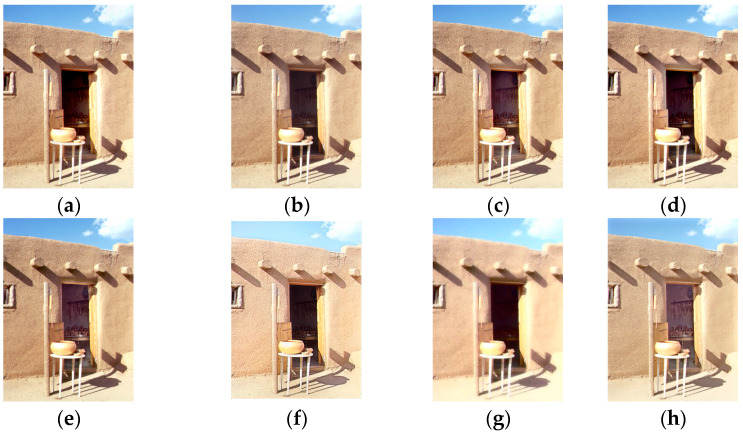
Comparison with other exposure fusion methods on the set ‘Door’. (**a**) MEF09 [4] (**b**) DSIFT [9] (**c**) DEM [10] (**d**) FMMEF [3], (**e**) MEF-Net [18] (**f**) DPE-MEF [12] (**g**) MEF-LUT [22] (**h**) MEF-CAAN.

Figure 14 shows the comparison results on the set ‘Door’. The results of MEF09 [4], DSIFT [9], FMMEF [3], DPE-MEF [12], and MEF-LUT [22] are significantly underexposed and loss of details inside the house. The wall surfaces of DPE-MEF [12] and MEF-LUT [22] are relatively overexposed and have lots of textures. The indoor scenes processed by MEF-Net [18] have a certain hue deviation.

For all the sets from Figure 11, Figure 12, Figure 13 and Figure 14 with two input images, our method recovers richer details and more natural colors compared with other methods.

In summary, our method achieves very high robustness in various complex scenes, whether fusing multiple input images or a pair of input images. The subjective observation shows that our method preserves more details and colors in both underexposed and overexposed areas and maintains high consistency with the real visual perception.

### 3.2. Objective Quality Measure Comparison

Our algorithm has achieved satisfactory results in a variety of complex scenes by subjective qualitative observation, and we also use the quality assessment metrics mentioned by Zhang [33] to evaluate our method quantitatively. As shown in Table 1, we evaluate the above eight MEF fusion methods using 10 standard metrics, including four types: information theory-based (EN [34] and NMI [35]), image feature-based (AG [36], EI [37], SD [38], and SF [39]), image structural similarity-based (Q^Y^ [40], and MEF-SSIM [15]), and human perception-inspired (Q^CB^ [41] and VIF [42]).

EN [34] measures the information contained in the fused image, and its larger value indicates that the fused image is more informative. NMI [35] is used to evaluate the information consistency between the fused image and the reference image, and a higher value indicates a better fusion. AG [36] is used to measure the clarity and detail performance of the fused image, where higher values indicate clearer edges and details of the fused image and better quality. EI [37] evaluates how much edge information is preserved and enhanced in the fused image, and its larger value indicates a higher quality of the fused image. SD [38] evaluates the contrast and detail richness of the fused image, and its larger value indicates a higher contrast of the fused image. SF [39] reflects the detail and clarity of the image by calculating the gradient change in the horizontal and vertical directions. The higher its value, the more detailed and edge information the image contains. Q^Y^ [40] is mainly based on the structural similarity (SSIM) and local contrast information of the image, which can effectively evaluate the performance of the fused image in detail preservation and contrast enhancement. MEF-SSIM [15] is based on the structural similarity index (SSIM) and extended and adjusted for the characteristics of multi-exposure image fusion, which can better reflect the human eye’s perception of image quality. Its larger value indicates higher fusion quality. Q^CB^ [41] evaluates the quality of the fused image by simulating the human eye’s perception of image details, contrast, and color. It combines multiple visual perception factors, including local contrast, brightness, and color information, to provide an integrated quality score. A larger value indicates a higher quality of fusion. VIF [42] is based on the properties of the Human Visual System (HVS) and measures image quality by comparing the information fidelity between the fused image and the reference image. The larger its value indicates the higher quality of the fused image.

Table 2 shows the average metric values of different MEF methods for all the test datasets. The NMI [35], EI [37], SD [38], and VIF [42] metrics of our method are the highest, and other metrics of the information theory-based, image features-based, and human perception-inspired metrics are also ranked among the top 3. This is mainly due to three advantages of our method. Firstly, our attention mechanism based method can focus on more important features and information of source images and has a stronger feature extraction ability than methods without attention mechanism. Secondly, our CAAN module expands the receptive field by dilated convolution and aggregates multi-scale context information without changing the image resolution so as to enlarge the global feature extraction boundary for better image modeling. Thirdly, our GFU module can make better use of the high-frequency information of the high-resolution input images. Therefore, our method has obvious advantages in feature richness, perception quality, and information preservation so as to achieve higher scores for the three types of metrics.

For the image structural similarity-based metrics Q^Y^ [40] and MEF-SSIM [15], the values of our result are higher than those of all three deep learning-based methods but lower than those of traditional methods because FMMEF [3] and DEM [10] adopt a certain strategy to increase MEF-SSIM [15] values. For example, the structural patch decomposition-based method FMMEF [3] calculates the weight map by decomposing the image block into mean intensity, signal intensity, and signal structure, which exactly customizes to improve MEF-SSIM [15]. DEM [10] adopts a special detail-enhancement component to increase MEF-SSIM [15] values specifically. DSIFT [9] adopts dense scale-invariant feature transform (SIFT) for fusion with the weight term-based scheme. The dense SIFT descriptor is used for local contrast extraction, which significantly increases Q^Y^ [40] values because Q^Y^ [40] just focuses on the SSIM and local contrast information. Meanwhile, color fidelity with respect to the radiance of real scenes is considered in DSIFT [9] to reduce color distortion, which is beneficial a lot to both local contrast and color information-sensitive metric Q^CB^ [41]. Unfortunately, one-sided increment of structural similarity, local contrast, or color in the above traditional methods has caused a decrease in other metrics, such as information theory-based metrics EN [34] and NMI [35], image feature-based metrics AG [36], EI [37], SD [38], SF [39], and the generic human perception-inspired metric VIF [42]. These traditional methods only use one or two metrics as quality evaluation, while our method adopts all four types of metrics for more comprehensive objective quality assessment, which is consistent with the results of the subjective evaluation.

### 3.3. Efficiency Comparison

We also compare the running time of our method with other deep learning-based methods and compute the average running time for representative testing sets, as shown in Table 3. For a fair comparison, we test all methods on the i7-12700 CPU. The parameters of DPE-MEF [12] are 13.6 M with a model size of up to 51.9 MB, which is much more than both MEF-Net [18] and MEF-CAAN. Our MEF-CAAN is much faster than DPE-MEF [12] and only a few milliseconds slower than MEF-Net [18] because the attention mechanism is introduced. The parameters of MEF-Net [18] and MEF-CAAN are 0.071 M and 0.074 M, and their model sizes are 0.33 MB and 0.35 MB, respectively. The parameters of the attention mechanism module are about 0.003 M, which proves that the attention mechanism has very low computational complexity. The lookup table-based method MEF-LUT [22] has a fast speed with a loss in fusion quality. Our method accomplishes feature extraction with low-resolution CAAN and adopts guided filtering for upsampling (GFU), which achieves high quality and efficiency.

### 3.4. Ablation Study

**Figure 15:**
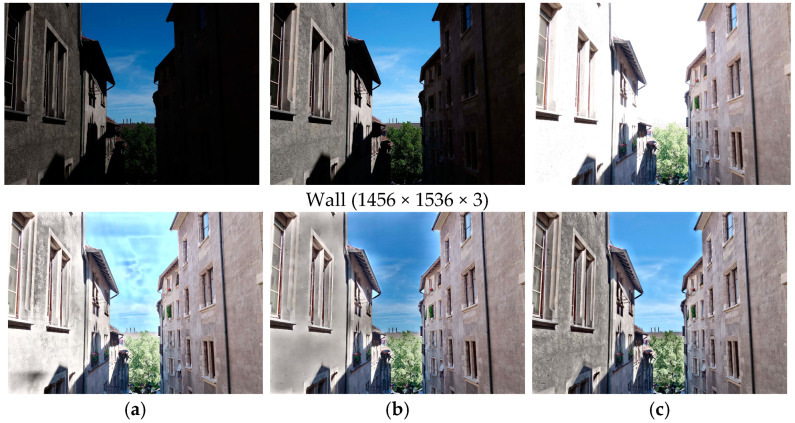
The fusion results in different attention mechanisms on the set ‘Wall’; (**a**) is the fusion result without an attention mechanism; (**b**) is the fusion result by adding the self-supervised attention mechanism; (**c**) is the fusion result by CAAN.

In this section, we conduct some kinds of ablation studies to prove the effectiveness of each module in our MEF-CAAN. Firstly, we trained our CAAN, the CAN network without channel attention, and the CAAN with the attention module replaced by the Transformer self-supervised attention module in STFNet [25]. The MEF-SSIM [15] results are listed in Table 4. We see that MEF-SSIM [15] scores have significant improvement after adding the attention mechanism. Besides, the network with our CAAN achieves slightly higher than the self-supervised attention module. We also show their fusion images comparison in Figure 15. There are halos and overexposed areas on the walls and in the sky for the network without an attention mechanism. For the fused image by self-supervised attention mechanism, the details and textures are lost on the walls. For the fused image by our CAAN module, both the sky and the walls are visually natural, and rich details are recovered. This demonstrates the effectiveness of our CAAN module.

Secondly, we perform an ablation study on the depth and width of the CAAN. The average MEF-SSIM [15] scores are listed in Table 5. The MEF-SSIM [15] scores increase as the depth and width of the network increase, as expected. It can also be observed that the MEF-SSIM [15] scores reach more than 0.970 with a depth of 6 or a width of 24 but do not improve significantly with more depth or width, so the depth and width of our network are set to 6 and 24 for higher efficiency performance.

**Figure 16:**
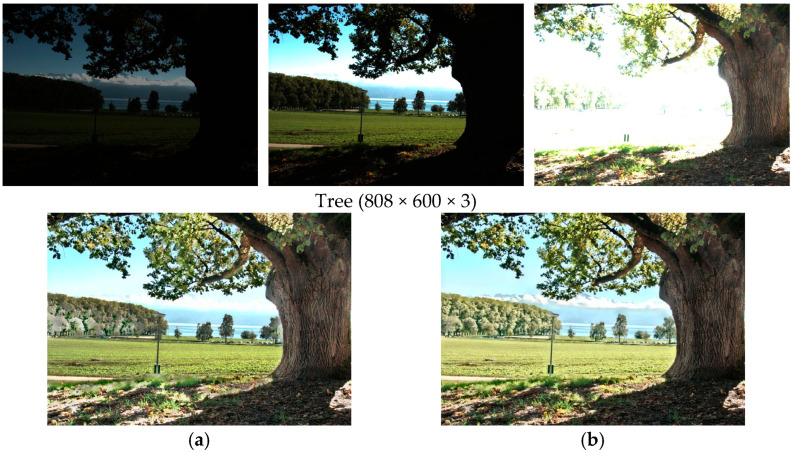
Fusion quality comparison of two upsampling methods for the test set ‘Tree’. (**a**) is the fusion result of simple bilinear upsampling. (**b**) is the fusion result of GFU.

Finally, we conduct an ablation study on the upsampling module, including our guided filtering for upsampling (GFU) and the simple bilinear upsampling. The MEF-SSIM [15] score of the method with simple bilinear upsampling is 0.9664, about 0.004 lower than that with GFU (0.9703). The results of different upsampling ways are shown in Figure 16. It can be seen that the high-frequency information is lost by the bilinear upsampling in Figure 16a. For example, the details of the woods and sky are blurred. The resulting image with our GFU preserves these details well because GFU uses the high-resolution input images as guide images, and the high-frequency details are well preserved. Therefore, this demonstrates the effectiveness of the GFU module.

### 3.5. Limitation in Dynamic Scenes

**Figure 17:**
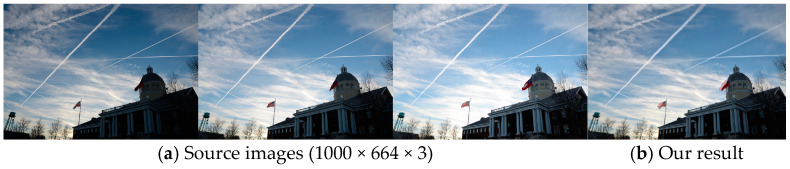
Fusion result of MEF-CAAN in dynamic scenes.

Our method aims to fuse images from static scenes but will cause ghost artifacts when dealing with dynamic scenes with moving objects. For example, there are obvious artifacts in the regions of flags, as shown in Figure 17b. It is challenging for the current framework to account for dynamic scenes mainly for the following two reasons; firstly, the current network lacks an effective motion detection mechanism to automatically correct pixel displacements across multi-exposure images. Secondly, the field of multi-exposure image fusion for dynamic scenes currently lacks reliable perceptual quality evaluation metrics or ground truths for supervision.

## 4. Discussion and Future Work

In this paper, we propose a multi-exposure image fusion network based on a context aggregation attention network (MEF-CAAN). The CAAN module expands the receptive field by dilated convolution, aggregates multi-scale context information without changing the image resolution, and expands the global feature extraction boundary; the channel attention mechanism introduced can focus on more important features and information of the source image so that the network can capture multi-scale information in the image and more features at the channel level. GFU module can better utilize the high-frequency information of high-resolution input images. Therefore, our method has obvious advantages in terms of feature richness, perceptual quality, and information retention. We conducted detailed experimental comparisons and ablation experiments to verify the effectiveness and rationality of our proposed method and demonstrate that our method has good time efficiency and robustness to various scenarios.

We hope to explore more effective detail enhancement strategies and incorporate them into our proposed method to obtain higher quality fused images. As our current MEF-CAAN is only applicable to static scenes, we also consider extending it to be suitable for dynamic scenes by introducing the ghost removal mechanism.

## Figures and Tables

**Table 1:** Evaluation metrics used in this paper.

Category	Name	Meaning
Information theory-based	EN [34]	Entropy
	NMI [35]	Normalized mutual information
Image feature-based	AG [36]	Average gradient
	EI [37]	Edge intensity
	SD [38]	Standard division
	SF [39]	Spatial frequency
Image structural similarity-based	Q^Y^ [40]	Yang’s metric
	MEF-SSIM [15]	Multi-Exposure Fusion Structural Similarity Index
Human perception-inspired	Q^CB^ [41]	Chen-Blum metric
	VIF [42]	Visual information fidelity

**Table 2:** Average metric values of different MEF methods for all the test datasets.

Metrics	Metric Values of Different Methods							
	MEF09 [4]	DSIFT [9]	DEM [10]	FMMEF [3]	MEF-Net [18]	DPE-MEF [12]	MEF-LUT [22]	MEF-CAAN
**EN** [34]	7.4236	7.4626	7.4714	7.4578	**7.5453**	7.3818	6.5017	7.5371 ^2^
**NMI** [35]	0.6049	0.6923	0.6992	0.5806	0.6937	0.6175	0.7740	**0.7817** ^1^
**AG** [36]	6.1537	5.8744	6.2253	6.2739	6.8229	**6.9472**	3.3768	6.8277 ^2^
**EI** [37]	60.0109	57.7305	60.9928	61.8744	69.8314	67.8608	32.8804	**71.4862** ^1^
**SD** [38]	54.0809	54.2094	57.7137	61.2351	62.2835	61.7648	54.7258	**63.7908** ^1^
**SF** [39]	20.2120	19.1154	20.4091	20.7031	22.8166	**23.3935**	12.1354	22.8113 ^3^
**Q^Y^** [40]	0.8618	**0.8966**	0.8892	0.8485	0.8269	0.7438	0.5282	0.8410 ^5^
**MEF-SSIM** [15]	0.9719	0.9753	0.9755	**0.9812**	0.9628	0.9479	0.8201	0.9703 ^5^
**Q^CB^** [41]	0.5137	**0.5200**	0.4951	0.4997	0.5032	0.4122	0.4665	0.5053 ^3^
**VIF** [42]	0.8122	0.7963	0.8495	0.9150	0.9381	0.7317	0.4156	**0.9416** ^1^

x ^y^ represents that x ranks y-th in each row. The value highlighted in bold indicates that it outperforms all the compared methods for the evaluation metric.

**Table 3:** Runing time with three deep learning-based methods for representative testing sets.

Sets	Running Time of Methods (s)			
	MEF-Net [18]	DPE-MEF [12]	MEF-LUT [22]	MEF-CAAN
Kluki	0.234	0.697	0.107	0.241
Lighthouse	0.249	0.594	0.096	0.249
Villa	0.246	0.682	0.088	0.247
Night	0.539	1.664	0.294	0.575
SevenElevenNight	3.556	10.864	1.868	3.750
Door	0.592	1.489	0.287	0.597
Average	0.903	2.665	0.457	0.943

**Table 4:** Average MEF-SSIM [15] scores for different attention mechanisms.

Attention Module	MEF-SSIM [15]
CAN without attention module	0.9604
Transformer self-supervised attention module	0.9693
CAAN module	0.9703

**Table 5:** Average MEF-SSIM [15] scores for different network depth and width. The default depth of our CAAN is set to 6, and the default width is set to 24.

Depth	4	5	6	7	8	9
MEF-SSIM	0.9661	0.9684	0.9703	0.9711	0.9716	0.9718
Width	8	16	24	32	48	64
MEF-SSIM	0.9578	0.9681	0.9703	0.9712	0.9718	0.9721

## Data Availability

The data that support the findings of this study are available from the corresponding author upon reasonable request.
